# Compassion fatigue and palliative care in neonatal nurses

**DOI:** 10.1017/S147895152400110X

**Published:** 2024-11-08

**Authors:** Fatma Bozdag, Oznur Basdas, Neslihan Atlı

**Affiliations:** 1 Health Sciences of Faculty -Department of Child Health and Diseases Nursing, Harran University, Sanlıurfa, Turkey; 2 Health Sciences of Faculty -Department of Child Health and Diseases Nursing, Erciyes University, Kayseri, Turkey; 3 Sanlıurfa Training and Research Hospital, Pediatric Emergency Clinic, Sanlıurfa, Turkey

**Keywords:** Compassion fatigue, neonatal intensive care, nurse, palliative, care

## Abstract

**Introduction:**

Neonatal intensive care units (NICUs) emerge as one of the areas where palliative care is most needed. This study was conducted to examine the attitudes and compassion fatigue levels of NICUs nurses working in Şanlıurfa, where the fertility rate and infant mortality are highest in Turkey, toward palliative care.

**Design:**

This study was conducted in descriptive type.

**Methods:**

The research was carried out with 204 (85%) nurses who agreed to participate in the research between October 2022 and February 2023, out of 240 neonatal intensive care nurses working in the NICU of 2 training and research hospitals and a university hospital in Şanlıurfa. The data of the study were collected using an Introductory Information Form, the Neonatal Palliative Care Attitude Scale, and the Compassion Fatigue Short Scale.

**Results:**

Nurses; compassion fatigue scale mean score was 61.46 ± 26.64, palliative care scale mean score was 3.13 ± 0.74 for organization subdimension, 2.85 ± 0.73 for resources subdimension, and 3.08 ± 0.89 for clinician subdimension. In the results of the study, 8 barriers (parents do not participate in decisions, there is not enough staff, lack of time to spend with the family, lack of policies/rules in institutions for palliative care, lack of education and communication, society’s beliefs, nurses’ personal attitudes toward death, and lack of appreciation of past experiences with palliative care) and 6 facilitators (Nurses’ ability to express their perceptions, views and beliefs about palliative care, to participate and support palliative care, to inform parents, to provide counseling, adequate physical conditions) for palliative care were determined.

**Conclusion:**

While it was determined that nurses had a slightly below moderate level of compassion fatigue and a close attitude toward organization and resources toward palliative care, it was determined that ethical conflict toward palliative care was high in clinical subdimension scores.

**Objectives and Significance of Results:**

It is recommended that all nurses working in the NICU obtain certificates, improvements in resources such as personnel and equipment, improvements in the shift work system and development of policies/rules in institutions for palliative care.

## Introduction

Despite the decrease in neonatal mortality rates due to scientific, medical, and technological developments, serious health problems are still observed in newborns due to reasons such as preterm birth, low birth weight, or congenital anomalies (Khraisat et al. 2023). Therefore, neonatal intensive care units (NICUs) emerge as one of the areas where palliative care is most needed (Esenay 2018). The World Health Organization supports the concept of palliative care in NICUs and emphasizes the need to provide care models that can achieve the best quality of life for newborns by controlling pain, and this concept is developing all over the world (Carter 2018; Maher-Griffiths 2022; Zhong et al. 2022). Although palliative care in NICUs has gained scientific ground with the palliative and end-of-life care for newborns and Infants guideline updated by the American National Association of Neonatal Nurses, there are currently no guidelines or practice protocols for neonatal palliative care in our country (Esenay 2018, Boan Pion et al. 2021).

In the palliative care team, which requires a multidisciplinary team working in harmony, nurses have an important place in the provision of palliative care services at all levels. Nurses working in NICUs have many important roles such as newborn care, pain assessment and management, and newborn and parent advocacy (Zhao et al. 2022). While performing their roles, nurses working in NICUs are affected by many conditions such as working conditions, lack of resources, stress, ethical dilemmas, and compassion fatigue (Asadollah et al. 2023; Fortney et al. 2020; Kim et al. 2019; Lewis 2019). These negative effects can facilitate palliative care, as well as create obstacles or conflicts, restricting palliative care.

A limited number of studies on palliative care have shown that it is affected by attitudes toward death, culture, education, previous experiences, and personal attitudes and perceptions (Abuhammad et al. 2023; Azzizadeh Forouzi et al. 2017; Cerratti et al. 2020; Sulun et al. 2021). On the other hand, it is known that compassion fatigue is more common in nurses working in areas where there are individuals with special needs (Kırcı and Kızıler 2021). For this reason, it is thought that the compassion fatigue that occurs in neonatal nurses who care for critically ill patients for extended periods may affect palliative care. This study was conducted to examine the attitudes and compassion fatigue levels of NICU nurses working in Şanlıurfa, where the fertility rate and infant mortality are highest in Turkey, toward palliative care (Turkish Statistical Institute, 2022).

*Research questions;*

- What is the compassion fatigue level of nurses working in the NICU and what are the factors affecting it?
- What is the palliative care attitude level of nurses working in the NICU and what are the factors affecting it?
- What is the relationship between the compassion fatigue of NICU nurses and their attitudes toward palliative care?
- What are the inhibiting and facilitating factors for neonatal nurses to provide palliative care?

## Methods

### Design and purpose of the study

The research was conducted as a descriptive study to determine the attitudes and compassion fatigue levels of nurses working in the NICU in Şanlıurfa toward palliative care.

### Population and sample of the study

The research population consisted of 240 neonatal intensive care nurses working in the NICUs of 2 training and research hospitals and the university hospital in Şanlıurfa. No sample selection was made in the study, and 204 (85%) NICU nurses who were not on leave between October 2022 and February 2023 when the study was conducted and agreed to participate in the study after the purpose of the study was explained, constituted the sample of the study.

### Data collection tools

In order to reach all nurses, repeated unit visits were carried out at specified intervals in cooperation with the nurses in charge of Neonatal Intensive Care in the hospitals constituting the population. The data of the study were collected with face-to-face questionnaires at the time period when the nurses were available and lasted an average of 15 minutes. Introductory Information Form, Neonatal Palliative Care Attitude Scale and Compassion Fatigue Short Scale were used to collect the data.

The *Introductory Information Form* included questions such as nurses’ age, sex, marital status, whether they had a child, educational status, the unit in which they worked, duration of work, and whether they had any certificates for neonatal intensive care or palliative care.

#### Neonatal Palliative Care Attitude Scale (NPCAS)

The Turkish validity and reliability study of the scale developed by Kain and Wilkinson was performed by Akay (Akay and Ozdemir 2021; Kain and Wilkinson 2013). The scale is used to determine factors that neonatal nurses see as obstacles and facilitators for palliative care practices. NPCAS is a 5-point Likert-type scale that includes items scored between strongly disagree (1) and strongly agree (5). Twelve items constituting the subdimensions of the 26-item scale are included in the scoring. The remaining 14 questions are used to evaluate nurses’ experiences with palliative care and nurses’ beliefs about infant death. The organization subdimension of the scale consists of items 5, 8, 15, 16, and 19, the resources subdimension consists of items 6, 7, 13, 14, and 24, and the clinical subdimension comprises items 20 and 21. There are no reverse-scored questions on the scale. A high score from the organization and resources subdimensions of the scale indicates a more positive attitude in these subdimensions, whereas a high score in the clinical subdimension indicates more moral and ethical conflicts related to the provision of neonatal palliative care. Internal consistency coefficients of the scale were 0.69 for the organization subdimension, 0.71 for the resources subdimension, and 0.68 for the clinical subdimension (Akay and Ozdemir 2021). In this study, the Cronbach alpha values were 0.65 for the organization subdimension, 0.61 for the resources subdimension, and 0.54 for the clinical subdimension.

#### Compassion Fatigue Short Scale (CFSS)

The scale, developed by Adams et al. in 2006, was adapted into Turkish by Dinç and Ekinci in 2019 (Adams et al. 2006; Dinc and Ekinci 2019). It is a 10-point Likert-type scale, scored between rarely/never (1) and very often (10), and includes 2 subdimensions of secondary trauma and occupational burnout. The secondary trauma subdimension consists of items c, e, h, j, and l, and the occupational burnout subdimension consists of items a, b, d, f, g, i, k, and m. The scale is scored between 13 and 130. As the scale scores increase, the level of compassion fatigue experienced by the participants also increases. The Cronbach alpha value of CFSS was found as 0.87 in the study of Dinç and Ekinci and was calculated as 0.92 in the present study (Dinc and Ekinci 2019).

### Data analysis

The IBM SPSS Statistics 24 statistical package program was used to evaluate the research data. Percentage values, arithmetic mean, standard deviation, median, minimum, and maximum values are given as descriptive statistics of the data. The Shapiro–Wilk normality test and Q–Q graphs were used to determine whether the data showed normal distribution. The independent samples t-test was used for comparisons of 2 independent groups, and one-way analysis of variance (ANOVA) was used for comparisons of more than 2 independent groups because the data were normally distributed. The statistical significance level was accepted as *p* < 0.05.

## Results

Introductory characteristics of the neonatal nurses are given in Table 1. It was determined that 31.4% of the neonatal nurses participating in the study were aged 25 years and under, 56.4% were female, 74.1% had a bachelor’s degree, 46.5% were married, and 32.4% had children. It was found that 62.3% of the nurses worked in the profession and 75.5% of them worked in the NICU for fewer than 5 years. It was determined that 41.7% of the nurses had NICU certificates and 8.3% had palliative care certificates (Table 1).

**Table 1.** Descriptive characteristics of neonatal nurses

Features	Number (*n*)	Percent (%)
Age (27.60 ± 3.81)		
25 age ↓	64	31.4
26–29 age	90	44.1
30 age ↑	50	24.5
Gender		
Female	115	56.4
Male	89	43.6
Education status		
High school	27	13.2
Associate degree	19	9.3
License degree	151	74.1
Postgraduate	7	3.4
Marital status		
Married	95	46.5
Single	107	52.5
Divorced	2	1.0
Having children		
Yes	66	32.4
No	138	67.6
Year of employment (4.90 ± 3.15)		
5 years ↓	127	62.3
6 years ↑	77	37.7
Years of employment in NICU (3.73 ± 2.73)		
5 years ↓	154	75.5
6 years ↑	50	24.5
Way of working		
Daytime	23	11.3
Shift/day and night	181	88.7
Having a newborn intensive care certificate		
Yes	85	41.7
No	119	58.3
Have a palliative care certificate		
Yes	17	8.3
No	187	91.7
Total	**204**	**100.0**

The mean CFSS score was 61.46 ± 26.64, and the mean NPCAS scores were 3.13 ± 0.74 for the organization subdimension, 2.85 ± 0.73 for the resources subdimension, and 3.08 ± 0.89 for the clinical subdimension (Table 2). The results showed that nurses had a slightly below moderate level of compassion fatigue and a good attitude toward organization and resources toward palliative care, and clinical subdimension scores showed that moral/ethical conflict toward palliative care was high.

**Table 2.** Compassion fatigue and palliative care scale scores of newborn nurses

Scales	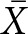 ±*SD*	*Med (Min–Max)*
Organization	3.13 ± 0.74	3 (1.20–4.80)
Resources	2.85 ± 0.73	2.8 (1–4.40)
Clinician	3.08 ± 0.89	3 (1–5)
Compassion Fatigue Scale	61.46 ± 26.64	59.5 (13– 127)

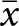
*: Mean, SD: standard deviation, Med: median, Min: minimum, Max: maximum.*

The numbers indicated above the variables were used to express the statistical difference within the group.

The CFSS and NPCAS scores of neonatal nurses according to their descriptive characteristics are given in Table 3. It was determined that the scores of CFSS were lower in nurses aged 25 and below, who were high school or graduate graduates, who were working daytime hours, and who had NICU certificates, and the difference between the groups was statistically significant (*p* = 0.001; *p* = 0.004; *p* = 0.033; and *p* = 0.007, respectively). It was determined that the clinical subscale scores of the female nurses in NPCAS were lower and the difference between the groups was statistically significant (*p* = 0.044). It was found that the variables of marital status, having a child, working years, working years in the NICU, experience of losing a newborn, and presence of a palliative care certificate did not affect the scores of CFSS and NPCAS (*p* > 0.05) (Table 3).

**Table 3.** Compassion fatigue and palliative care scale scores according to the descriptive characteristics of neonatal nurses

		Palliative Care Scale		
	Compassion Fatigue Scale	Organization	Resources	Clinician
Features	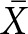 ±*SD*	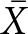 ±*SD*	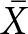 ±*SD*	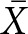 ±*SD*
Age				
25 age^1^	51.30 ± 23.31	3.21 ± 0.78	2.85 ± 0.79	3.06 ± 0.90
26−29 age^2^	66.60 ± 26.28	3.11 ± 0.71	2.85 ± 0.76	3.05 ± 0.89
30 age ↑^3^	65.20 ± 28.09	3.09 ± 0.76	2.86 ± 0.63	3.16 ± 0.93
*Test (F; p)*	***7.246; .001 (1 < 2 = 3)***	*.464; .629*	*.005; .995*	*.258; .773*
Gender				
Female	60.79 ± 27.05	3.05 ± 0.77	2.80 ± 0.72	2.97 ± 0.93
Male	62.31 ± 26.23	3.25 ± 0.69	2.93 ± 0.76	3.22 ± 0.85
*Test (t; p)*	*−.404; .686*	*−1.954; .052*	*−1.211; .227*	***−2.023; .044***
Education status				
High school^1^	46.78 ± 25.19	3.02 ± 0.69	2.97 ± 0.66	3.07 ± 0.81
Associate degree^2^	70.32 ± 27.34	3.44 ± 0.66	3.04 ± 0.69	3.11 ± 0.99
License degree^3^	63.56 ± 26.22	3.11 ± 0.74	2.79 ± 0.75	3.09 ± 0.90
Postgraduate^4^	48.57 ± 17.53	3.37 ± 1.09	3.29 ± 0.65	2.79 ± 1.07
*Test (F; p)*	***4.517; .004 (1 = 4 < 2 = 3)***	*1.589; .193*	*1.831; .143*	*.262; .853*
Way of working				
Daytime	50.30 ± 20.92	3.02 ± 0.77	2.83 ± 0.79	3.07 ± 0.80
Shift/day and night	62.87 ± 27.00	3.15 ± 0.74	2.86 ± 0.73	3.08 ± 0.91
*Test (t; p)*	***−2.150; .033***	*−.834 .405*	*−.199 .843*	*−.088 .930*
Having a newborn intensive care certificate				
Yes	55.54 ± 22.19	3.05 ± 0.72	2.87 ± 0.76	3.14 ± 0.89
No	65.68 ± 28.76	3.21 ± 0.75	2.85 ± 0.73	3.04 ± 0.91
*Test (t; p)*	***−2.722; .007***	*−1.502 .135*	*.218 .828*	*.729 .467*

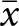
*: Mean, SD: standard deviation. Independent samples t and one-way ANOVA tests were used.*

The correlation between CFSS and NPCAS scores is given in Table 4. It was found that there was a weak negative correlation between the CFSS score and NPCAS’s resources subdimension score (*p* = 0.002) (Table 4).

**Table 4.** Correlation of newborn nurses’ compassion fatigue and palliative care scale scores

	Compassion Fatigue Scale	
Scales	*r*	*p*
Organization	*−.051*	*.466*
Resources	−.212**	*.002*
Clinician	*−.019*	*.788*

The CFSS and NPCAS scores and their responses to NPCAS questions are given in Table 5. It was determined that there were 8 barriers and 6 facilitators for palliative care. The facilitators, barriers, and contradictory responses stated by nurses for palliative care were as follows. ***Facilitators*** were the nurses’ ability to express their perceptions, views, and beliefs about palliative care, their participation and support in palliative care, informing parents, providing counseling, and adequate physical conditions. ***Obstacles*** were parents not participating in decisions, insufficient numbers of staff, not enough time to spend with the family, lack of policies/rules in institutions for palliative care, lack of education and communication, beliefs of the society, nurses’ personal attitudes toward death, and not being appreciated regarding past experiences of palliative care. ***Contradictions*** were perceptions of nurses and society toward palliative care and curative care (Table 5).

**Table 5.** Neonatal nurses’ attitude toward barriers to NPC

Subscales	Items	Strongly disagree/disagree, *n* (%)	Unsure, *n* (%)	Strongly agree/agree, *n* (%)
Organization	5. The medical staff support palliative care for dying babies in my unit	49 (24%)	39 (19.1%)	116 (56.9%)
	8. In my unit, parents are involved in decisions about their dying baby	96 (47.1%)	45 (22.0%)	63 (30.9%)
	15. In my unit, when a diagnosis with a likely poor outcome is made, parents are informed of palliative care options	58 (28.4%)	48 (23.5%)	98 (48.1%)
	16. In my unit the team expresses its opinions, values and beliefs about providing care to dying babies	52 (25.5%)	43 (21.1%)	109 (53.4%)
	19. All members of the health care team in my unit agree with and support palliative care when it is implemented for a dying baby	69 (33.8%)	43 (21.1%)	92 (45.1%)
Resources	6. The physical environment of my unit is ideal for providing palliative care to dying babies	70 (34.3%)	37 (18.1%)	97 (47.6%)
	7. My unit is adequately staffed for providing the needs of dying babies requiring palliative care and their families	95 (46.6%)	32 (15.7%)	77 (37.7%)
	13. When a baby dies in my unit, ı have sufficient time to spend with the family	116 (56.9%)	48 (23.5%)	40 (19.6%)
	14. There are policies/guidelines to assist in the delivery of palliative care in my unit	67 (32.8%)	62 (30.4%)	75 (36.8%)
	24. When a baby dies in my unit, counseling is available if ı need it	67 (32.8%)	59 (28.9%)	78 (38.3%)
Clinician	20. In my unit, the staff go beyond what they feel comfortable with in using technological life support	68 (33.3%)	52 (25.5%)	84 (41.2%)
	21. In my unit, staff are asked by parents to continue life-extending care beyond what they feel is right	63 (30.9%)	60 (29.4%)	81 (39.7%)
Experiences and Attitudes	1. Palliative care is as important as curative care in the neonatal environment	12 (5.9%)	18 (8.8%)	174 (85.3%)
	2. I have had experience of providing palliative care to dying babies and their families	52 (25.5%)	33 (16.2%)	119 (58.3%)
	3. I feel a sense of personal failure when a baby dies	109 (53.4%)	24 (11.8%)	71 (34.8%)
	4. There is support for neonatal palliative care in society	51 (25%)	69 (33.8%)	84 (41.2%)
	9. My previous experiences of providing palliative care to dying babies have been rewarding	88 (43.1%)	53 (26%)	63 (30.9%)
	10. When babies are dying in my unit, providing pain relief is a priority for me	36 (17.6%)	32 (15.7%)	136 (66.7%)
	11. I am often exposed to death in the neonatal environment	85 (41.7%)	18 (8.8%)	101 (49.5%)
	12. Palliative care is necessary in neonatal nursing education	23 (11.3%)	25 (12.3%)	156 (76.4%)
	17. Caring for dying babies is traumatic for me	99 (48.5%)	40 (19.6%)	65 (31.9%)
	18. I have received in-service education that assists me in supporting and communicating with parents of dying babies	122 (59.8%)	32 (15.7%)	50 (24.5%)
	22. My personal attitudes about death affects my willingness to deliver palliative care	59 (28.9%)	55 (27%)	90 (44.1%)
	23. Palliative care is against the values of neonatal nursing	137 (67.2%)	40 (19.6%)	27 (13.2%)
	25. There is a belief in society that babies should not die, under any circumstances	74 (36.3%)	50 (24.5%)	80 (39.2%)
	26. Curative care is more important than palliative care in the neonatal intensive care environment	61 (29.9%)	56 (27.5%)	87 (42.6%)

## Discussion

This study is the first to evaluate the relationship between palliative care and compassion fatigue in NICU nurses. The importance of the study lies in the fact that it was conducted in a province in Turkey where fertility rates and infant mortality were ranked second highest. It is known that compassion fatigue is affected by many factors such as care burden, age, and education level (Kesbic and Boz 2022; Richardson and Greenle 2020). Similarly, in the present study, it was determined that age, education level, working system (day or night), and having a newborn intensive care certificate affected compassion fatigue (Table 3). In addition, it was determined that there was a weak negative correlation between compassion fatigue and the resources subdimension of NPCAS (*p* = 0.002, Table 4). Although this relationship was weak, it was very important. It is known that compassion fatigue in nurses causes a decrease in the quality of care provided (Asadollah et al. 2023; Wong et al. 2023).

It was accepted by nurses that the quality of palliative care in NICUs should be improved (Ferrell et al. 2020). In a review study examining the attitudes of neonatal nurses toward palliative care, it was reported that many situations such as lack of education on palliative care, physical environment, technical requirements, belief in palliative care, negative attitude, discomfort arising from the use of life support, pressures of parents, the perspective of society, the feeling of trauma in the caregiver of dying baby, and the sense of personal failure in nurses were obstacles (Abuhammad et al. 2023). To increase the effectiveness of palliative care programs, it is a priority to provide training to neonatal nurses on palliative care (Chin et al. 2021). In the present study, it was determined that very few nurses had palliative care certificates (Table 1), and the fact that the majority of nurses stated that palliative care should be included in neonatal nursing education (item 12) and that palliative care was not against the values of neonatal nursing (item 23) showed that they had a positive perception toward education (Table 5). Similarly, in other studies conducted in Turkey, it was stated that most of the nurses working in NICUs did not receive training in palliative care (Erel and Buyuk 2021; Girgin et al. 2022). Again, the results of studies conducted in different countries [China, the United States of America (USA), and Saudi Arabia] showed that nurses were insufficient in determining the transition process to palliative care, they did not receive training on palliative care, they did not find the education they received in the field of education sufficient, and they thought that the cultures of the societies should be taken into account in the training programs to be created (Gu et al. 2022; Khraisat et al. 2023; Wright et al. 2011). Country-specific standardized neonatal palliative care protocols should be established and in-house training should be conducted by adhering to these principles (Abuhammad et al. 2023; Kyc et al. 2020). According to their answers, 30.4% of the nurses had no idea about the existence of policies/rules prepared to help practice palliative care in their institution, 32.8% knew that there were policies/rules, and 36.8% stated that there were no prepared policies/rules (item 14; Table 5). These results show that nurses are not aware of the policies and rules of their institutions and evaluate them inadequately. Parallel to the results of the research, in a study conducted in Brazil, nurses working in NICUs stated that there was inconsistency in institutional policies for palliative care, that there were no standardized palliative care protocols, and that in-service training was insufficient, arguing that these factors were obstacles to palliative care (De Oliveira et al. 2018).

During the neonatal palliative care process, nurses both care for dying babies and inform the parents about the worsening prognosis (Camilo et al. 2022). For this reason, nurses should be able to inform parents about palliative care options, have enough time to stay in contact with parents throughout the process, include them in the decision-making process, and have the physical conditions to do all these (Banazadeh and Rafii 2021; Chin et al. 2021). Most of the nurses stated that the physical conditions of the unit were ideal for providing palliative care for dying infants (item 6), that the medical staff supported the palliative care of dying infants (item 5), that relieving the pain of dying infants was the priority (item 10), that when the infant was diagnosed as having a poor-prognosis disease, the parents were informed about palliative care options (item 15), that they could express their opinions and beliefs about caring for dying babies (item 16), that they could provide counseling if necessary when the baby died (item 24), and that they participated and supported care when palliative care would be provided for a dying baby (item 19). These statements show that nurses working in NICU have a positive perception toward palliative care and perceive these factors as facilitating palliative care. Similarly, studies conducted in Turkey and the USA report that nurses’ attitudes toward palliative care are positive and that they support palliative care (Girgin et al. 2022; Kachlová and Bužgová 2021; Kyc et al. 2020). On the other hand, the majority of nurses stated that there were insufficient staff to provide palliative care in their institutions (item 7), that parents did not participate in decisions about their dying babies (item 8), that there was not enough time to spend with the family (item 13), and that there was not enough training to support and communicate with parents (item 18) (Table 5). Similarly, studies conducted in Italy, Iran, Taiwan, and the USA reported that inadequate physical conditions, limited staff, and limited time to spend with family were obstacles to palliative care (Azzizadeh Forouzi et al. 2017; Cerratti et al. 2020; Chen et al. 2013; Kyc et al. 2020; Wright et al. 2011).

Palliative care was also affected by the expectation of parents, the beliefs of nurses, and the perspective and culture of society (Kim et al. 2019). The majority of nurses stated that they were able to express their opinions and beliefs about caring for dying babies (item 16), that they did not feel a sense of personal failure when a baby died (item 3), that society supported neonatal palliative care (item 4), and that caring for dying babies was not traumatic for them (item 17). On the other hand, they stated that their previous experiences with palliative care were not appreciated (item 9), that their personal attitudes toward death affected their willingness to provide palliative care (item 22), and that there was a belief in society that babies should not die under any circumstances (item 25), which were the contradictory responses (Table 5). In a study conducted in Italy, it was found that nurses could not share their personal views on palliative care and families were generally not aware of neonatal palliative care options because they were not informed (Cerratti et al. 2020). Similarly, in studies conducted in Taiwan and the USA, it was determined that nurses’ inability to express their views, values, and beliefs about palliative care, the social belief that babies should not die, parents’ wishes, and lack of communication were obstacles to palliative care (Chen et al. 2013; Kyc et al. 2020; Wright et al. 2011). When the literature results are reviewed, it is seen that these factors negatively affect palliative care and are among the obstacles to providing it effectively.

Most of the nurses agreed that palliative care was as important as curative care (item 1) and that curative care was more important than palliative care (item 26) (Table 5). Likewise, in another study conducted in China, nurses gave contradictory answers by agreeing with these 2 items at a high rate (Gu et al. 2022). Negative traumatic experiences of nurses, cultural problems, or attitudes and beliefs about palliative care may cause nurses to give contradictory answers.

### Strengths and limitations

This study is the first to evaluate the relationship between palliative care and compassion fatigue in NICU nurses. In addition, the research was conducted in Şanlıurfa, where the birth rate and infant mortality rate are the second in Turkey. The fact that most of the nurses do not have newborn and palliative care certificates and that their working years in the NICU are less than 5 years can be counted among their weaknesses. The experiences of participants who took the survey may not entirely mirror those of those who did not. Also, the results is constrained by the fact that this study was restricted to a single city located in Türkiye. And lastly, using a self-report questionnaire may create bias since participants might not always provide accurate accounts of their experience.

### Conclusion and recommendations

The results showed that nurses had a slightly below moderate level of compassion fatigue and a good attitude toward organization and resources toward palliative care, and the clinical subdimension scores showed that moral/ethical conflict toward palliative care was high. It was found that there was a weak negative correlation between the neonatal nurses’ compassion fatigue and palliative care scale resources subdimension scores. In line with the results of the research, different from the literature, when the facilitators and obstacles were examined, it was determined that the nurses’ past experiences of palliative care, and not being appreciated and rewarded, were obstacles. It is understood that palliative care is still not as important as curative care and awareness is not formed among health professionals working in NICUs. It is recommended that regular palliative care programs that address culture-specific issues and communication skills should be integrated into institutions to improve and increase the visibility of neonatal palliative care.

### Implications for clinical practice

The results of this research can be used in clinical practice to identify potential areas of improvement in addressing compassion fatigue and moral/ethical conflicts among nurses working in palliative care settings. By understanding that younger nurses, those with lower education levels, and those working specific shifts or with certain certifications may be at higher risk for compassion fatigue, healthcare organizations can tailor interventions and support programs to better meet the needs of these individuals. Additionally, the finding that nurses have a good attitude toward organization and resources toward palliative care suggests that focusing on enhancing resources and support systems within the organization could help mitigate the negative impacts of compassion fatigue and moral/ethical conflicts. This could involve providing additional training, resources, and support for nurses working in palliative care, as well as creating a culture of open communication and support within the workplace. Overall, the results of this research provide valuable insights that can guide healthcare organizations in developing targeted interventions to address compassion fatigue and moral/ethical conflicts among nurses in palliative care settings, ultimately improving the quality of care provided to patients and enhancing the well-being of healthcare professionals.

## Supporting information

Bozdag et al. supplementary material Bozdag et al. supplementary material

## Supplementary material

The supplementary material for this article can be found at https://doi.org/10.1017/S147895152400110X.
