# Supplementary material for: Compassion fatigue and palliative care in neonatal nurses
Source: Palliat Support Care. 2024 Nov 8;23:e4. doi: 10.1017/S147895152400110X (PMC13168775; doi:10.1017/S147895152400110X)
Supplement: Bozdag et al. supplementary material [file S147895152400110Xsup001.docx]

**COMPASSION FATIGUE AND PALLIATIVE CARE IN NEONATAL NURSES**

**Short Title:** Care and fatigue in neonatal nurses

**Fatma BOZDAG^1^, Oznur BASDAS^2^, Neslihan ATLI^3^**

1. **Fatma BOZDAG**, Lecturer Doctor

**ORCID ID:** 0000-0002-2636-8992

Health Sciences of Faculty -Department of Child Health and Diseases Nursing, Harran University, Sanlıurfa, Turkey-63000.

**Phone:** +90 541 775 27 08; +90414 3183000-2373

**E-mail:** [fatmabozdag@harran.edu.tr / fatosbozdag42@gmail.com](mailto:fatmabozdag@harran.edu.tr%20/%20fatosbozdag42@gmail.com)

1. **Oznur BASDAS,** Associate Professor (**Corresponding author**)

**ORCID ID:** 0000-0003-0752-6614

Health Sciences of Faculty -Department of Child Health and Diseases Nursing, Erciyes University, Kayseri, Turkey-38010.

**Phone:** +905462441012; +903522076666- 28568

**E-mail:** [obasdas@erciyes.edu.tr](mailto:obasdas@erciyes.edu.tr)

1. **Neslihan ATLI**, PhD

**ORCID ID:**0000-0002-0973-8867

Sanlıurfa Training and Research Hospital, Pediatric Emergency Clinic, Sanlıurfa, Turkey- 63250.

**Phone:** +905456330214

**E-mail:** [neslihanatli96@gmail.com](mailto:neslihanatli96@gmail.com)

**CONFLICT OF INTEREST**

There are no financial conflicts of interests.
